# Allergic rhinitis: the eligible candidate to mite immunotherapy in the real world

**DOI:** 10.1186/s13223-017-0185-x

**Published:** 2017-02-21

**Authors:** Giorgio Ciprandi, Valentina Natoli, Paola Puccinelli, Cristoforo Incorvaia, Italian Cometa Study Group, S. Barberi, A. Foresi, E. Galli, F. Gani, M. Gelardi, I. Lamantia, D. Peroni, E. Ridolo, G. E. Senna, L. Ricciardi, A. Romano, O. Rossi, G. Scala, S. Testi

**Affiliations:** 1 Department of Internal Medicine, Istituto di Ricovero e Cura a Carattere Scientifico (I.R.C.C.S.), Azienda Ospedaliera Universitaria San Martino-IST, Largo R. Benzi 10, 16132 Genoa, Italy; 2 Scientific, Pharmacovigilance and Regulatory Department Stallergenes, Milan, Italy; 3 Allergy/Pulmonary Rehabilitation Unit, Istituti Clinici di Perfezionamento Hospital, Milan, Italy

**Keywords:** Allergen immunotherapy, House dust mite allergy, Allergic rhinitis, Asthma, Real life

## Abstract

As standard drug treatment of allergic rhinitis (AR) is not completely satisfactory, allergen immunotherapy (AIT) represents the only current treatment with the potential to modify the natural history. House dust mite (HDM) allergy is very common. The aim of the current experience was to describe the clinical profile of HDM-allergic patients with AR who received AIT in a real world model, such as allergy clinics. Globally, 239 patients (126 adults and 113 children; 107 females and 132 males; mean age 21 years, age range 6–56 years) were evaluated. AIT was prescribed in 59 patients (24.7%), 44 adults (35%) and 15 children (13.3%). The current findings deriving from this real world multicentre study are consistent with previous investigations on HDM-AIT and define some clinical characteristics of the eligible candidate to this treatment. In fact, severity of ocular-nasal symptoms and over-use of symptomatic medications may typify the ideal candidate to HDM-AIT and SLIT was the preferred choice.

Allergic rhinitis (AR) is a common illness that impairs quality of life and causes substantial economic burden [1]. There is evidence that AR may affect up to 40% of the general population [2]. Furthermore, the worldwide prevalence of AR is increasing. AR causes significant morbidity to affected individuals and has been estimated to account for relevant lost school or work days per year. Moreover, AR is a risk factor for asthma onset and worsening.

As standard drug treatment of AR is not completely satisfactory, allergen immunotherapy (AIT), either subcutaneous immunotherapy (SCIT) or sublingual  immunotherapy (SLIT), represents the only current treatment with the potential to modify the course of allergic respiratory diseases by producing immunological and clinical tolerance to the causal allergen [3]. In this regard, allergy to house dust mites (HDM) is a very common cause of AR, mainly in children. There is some evidence that AIT to HDM is effective [4], but the findings are still conflicting. As AIT is a therapy characterized by long duration, expensive cost, and sometimes limited efficacy, there is the need to well define the characteristic of the ideal eligible candidate to HDM-AIT as recently appointed [5]. Karaman and colleagues performed a study aiming to investigate potential parameters useful in predicting the clinical response to AIT in HDM allergic children with asthma [6]. The authors enrolled 107 children mono-allergic to HDM: 47 were treated with a 4–5 year course of SCIT, 67 were treated with medications alone and served as control. The only parameter predictive of a clinical response to AIT was the baseline serum total IgE level.

We believe that the issue concerning the definition of the ideal candidate to AIT is clinically relevant and deserves adequate consideration. In this regard, we report the outcomes provided by an Italian multi-centre observational survey conducted in 17 allergy clinics. The aim of the study was to describe the clinical profile of HDM-allergic patients with AR who were allocated to AIT in a real world model, such as allergy clinics. Inclusion criteria were: age between 6 and 60 years, mono-allergy to HDM, and written informed consent. Exclusion criteria were: different age, allergy to other allergens, previous AIT, severe psychiatric disorders. The study was approved by the Ethical Committee of each participating allergy centre. The study was managed, monitored, and analysed by a Contract Research Organization (CD-Pharma Group, Milan, Italy) using electronic case report forms. Stallergenes Italia (Milan, Italy) sponsored the study.

Considered parameters were: AR duration, symptom periodicity, symptom severity perception assessed by visual analogue scale (VAS), asthma comorbidity, use of antihistamines and topical corticosteroids. χ^2^ test (with Fisher’s correction) and logistic regression were used (SAS system version 9.4) to analyze the data. Two hundred thirty-nine patients (126 adults and 113 children; 107 females and 132 males; mean age 21 years, age range 6–56 years) were evaluated. All mite-allergic patients presented to clinic with AR symptoms. AIT was prescribed in 59 patients (24.7%), 44 adults (35%) and 15 children (13.3%). AIT prescription was decided considering the symptom burden, the medication use, and the willingness of patients.

The patients’ symptoms, although perennial, had a typical seasonal trend with an evident worsening during the fall-winter period (namely from October to March). This finding is consistent with previous studies that pointed out a periodicity of HDM presence at home, symptoms, drug use, and inflammatory features [7, 8]. Moreover, this finding might deserve interesting debate about the HDM-AIT schedule as recently argued [5].

AIT was prescribed only in about 25% of all screened patients, particularly only in 13% of paediatric subjects and about in 1/3 of adults. This outcome is quite surprising because the main inclusion criterion was mono-allergy to HDM, that is the optimal feature to indicate AIT. It is obvious that many factors may affect AIT prescription, including allergists belief, patient persuasion, AIT duration, costs, misconceptions, etc. Concerning the route of administration of AIT, SLIT was prescribed in 98% of patients. A mixture of *Dermatophagoides farinae* and *D. pteronyssinus* allergen extracts was used in 95% of prescriptions.

About the factors affecting the AIT choice, a series of parameters were evaluated. AR duration was longer in AIT-treated group than in other patients (7.2 vs. 6 years). Asthma was present in 41% of AIT-treated patients and in 34% of remaining patients. Interestingly, cough was the most relevant asthma symptom reported in AIT group, whereas breathlessness was most frequent in AIT-untreated patients. The severity of both conjunctival and nasal symptom perception was greater in AIT-treated patients. VAS for ocular symptoms were 3.95 and 2.87 respectively (p = 0.01); VAS for nasal symptoms was 7.9 and 5.9 respectively (p = 0.0001). Consistently, conjunctivitis co-morbidity was more frequent in AIT-treated group (p = 0.002) as well as nasal polyps (p = 0.02).

The significant predictive factors were: use of antihistamines (OR 1.3; CI 1.03–1.31; p = 0.01), use of topical corticosteroids (OR 1.3; CI 1.02–1.29; p = 0.02), and rural residence (OR 4.4; CI 1.32–15.1; p = 0.02). These outcomes underline that the patient candidate to HDM-AIT presents a clinical feature quite different from other HDM patients. Obviously, the present study was performed in real life and some factors might interfere with results. For example, the above considered aspects (e.g. duration, cost, etc.) may influence the rate of AIT prescription. However, it is apparent that AIT candidates generally present a more severe clinical profile, mainly concerning ocular and nasal symptom and medication use. Therefore, AIT was preferentially prescribed in patient with moderate-to-severe AR. Noteworthy, this aspect is consisting with the evidence that AIT is more effective in patients complaining more bothersome symptoms. In fact, a study reported that patients with severe symptoms were more responsive to AIT than patients with mild symptoms [9]. Rural residence is a singular outcome, that probably might be correlated with greater humidity present in country areas. Moreover, there is evidence that urban children may be exposed to protective factors so experiencing potentially milder HDM-AR [10].

In conclusion, the current findings deriving from this real world multicentre study are consistent with previous investigations on HDM-AIT [4], and define some clinical characteristics of the eligible candidate to this treatment. In fact, severity of ocular-nasal symptoms and over-use of symptomatic medications may typify the ideal candidate to HDM-AIT and SLIT was the preferred choice.

## Abbreviations

AIT: allergen immunotherapy
HDM: house dust mite
AR: allergic rhinitis
SLIT: sublingual immunotherapy
VAS: visual analogue scale
